# Increased efficacy of PARP inhibitors against cisplatin-sensitive and -resistant ovarian cancer cells mediated via ATR and ATM inhibition

**DOI:** 10.1038/s41420-025-02740-1

**Published:** 2025-10-06

**Authors:** Philipp König, Leon Bade, Julia Maria Eichhorn, Helena Skalski, Jindrich Cinatl, Martin Michaelis, Gerd Bendas

**Affiliations:** 1 Department of Pharmacy, University Bonn, Bonn, Germany; 2 Institute of Medical Virology, University Hospital Frankfurt, Goethe University, Frankfurt am Main, Germany; 3 Interdisciplinary Laboratory for Paediatric Tumour and Virus Research, Dr Petra Joh Research Institute, Frankfurt am Main, Germany; 4 School of Natural Sciences, University of Kent, Canterbury, Kent UK

**Keywords:** DNA damage response, Ovarian cancer, Cancer therapeutic resistance, Cancer microenvironment

## Abstract

PARP inhibitors (PARPi) are approved for the treatment of platinum-based therapy-responsive ovarian cancer. However, this severely restricts their therapeutic potential, since there is only limited knowledge on the efficacy of PARPi in platinum drug-resistant ovarian cancer cells. Here, we studied three approved PARPi, niraparib, olaparib, and rucaparib in three ovarian cancer cell lines and their cisplatin-resistant sublines. Complex response profiles demonstrated that cisplatin resistance was not consistently associated with cross-resistance to PARPi. The combination of PARPi with inhibitors of relevant DNA damage response kinases which are potentially involved in PARPi resistance, such as ATR, ATM, CHK1, and WEE1 again resulted in complex activity patterns, but also identified ATR and ATM as the most promising targets for increasing PARPi activity. Cell adhesion-mediated resistance via collagen I is known to mediate cisplatin resistance. Here, we show that collagen I can also mediate PARPi resistance, which can also be tackled by ATR and ATM inhibition in cisplatin-sensitive and cisplatin-resistant ovarian cancer cell lines. In conclusion, our findings revealed complex, cell line-specific PARPi response profiles. This complexity is in line with other studies investigating drug-resistant cancer cell lines and with the complex evolutionary processes in tumors from cancer patients. Notably, cisplatin resistance was not directly correlated with PARPi resistance, and ATM and ATR inhibitors can increase PARPi activity against cisplatin-sensitive and -resistant ovarian cancer cells. Moreover, we demonstrated for the first time that cell adhesion-mediated resistance can contribute to PARPi resistance, which can also be alleviated by ATR and ATM.

## Introduction

PARP-inhibitors (PARPi) are approved for the maintenance therapy of platinum-sensitive ovarian cancer. PARP1 binds to DNA single-strand breaks and generates poly(adenosine diphosphate ribose) (PAR) chains. This process, known as PARylation, initiates the repair of DNA single-strand breaks. PARP1 inhibition prevents this single-strand break repair, resulting in the formation of DNA double-strand breaks and the activation of homologous recombination as DNA repair mechanism. BRCA1/2-mutant cancer cells are defective in homologous recombination and particular sensitive to PARP inhibition. Moreover, PARP1-mediated auto-PARylation is required to separate PARP1 from repaired DNA. PARPi also inhibit this auto-PARylation trapping PARP1 on the DNA and stalling the replication fork [1–3].

While the PARPi olaparib and rucaparib have only been approved for the treatment of BRCA1/2-mutant tumors [4, 5], niraparib is also approved for BRCA wild-type tumors [6]. Currently, PARPi are only used for ovarian cancer that responds to platinum-based standard treatment [7]. Hence, the occurrence of platinum drug resistance, a major reason for the failure of ovarian cancer therapies, is currently a contraindication for PARPi treatment in ovarian cancer. However, there are only a few studies and limited knowledge on the efficacy of PARPi against platinum-resistant cancer [8–10].

One of the most important cell mechanisms to resist platinum toxicity is an enhanced capacity of DNA-repair processes [11, 12]. Among the various approaches to overcome platinum-based resistances of tumors, inhibitors of the DNA damage response (DDR) pathway offer a promising way, which has also been tested in combination with some PARPi [13–16].

Cell adhesion-mediated drug resistance (CAM-DR) describes the phenomenon that cancer cell resistance can be mediated by cell binding to other cells or components of the extracellular matrix (ECM) [17]. For example, the ECM constituent collagen I was demonstrated to induce cisplatin-resistance in cancer cells [18]. Additionally, there is evidences that collagen-induced signaling can prevent DNA damage and mediate DNA repair [19, 20]. However, the impact of CAM-DR on the efficacy of PARPi is not known.

To address these knowledge gaps, we here investigated the effects of the PARPi niraparib, olaparib, and rucaparib in a panel of ovarian cancer cell lines and their cisplatin-resistant sublines. Notably, cisplatin resistance was not automatically associated with PARPi resistance, and ATR and ATM were identified as promising therapeutic targets for the sensitization of ovarian cancer cells to PARPi. Moreover, we found that collagen I can also mediate resistance to PARPi and that ATR and ATM inhibition interferes with the collagen I-mediated resistance.

## Results

### Varying PARPi response profiles among ovarian cancer cell lines

**Fig. 1:**
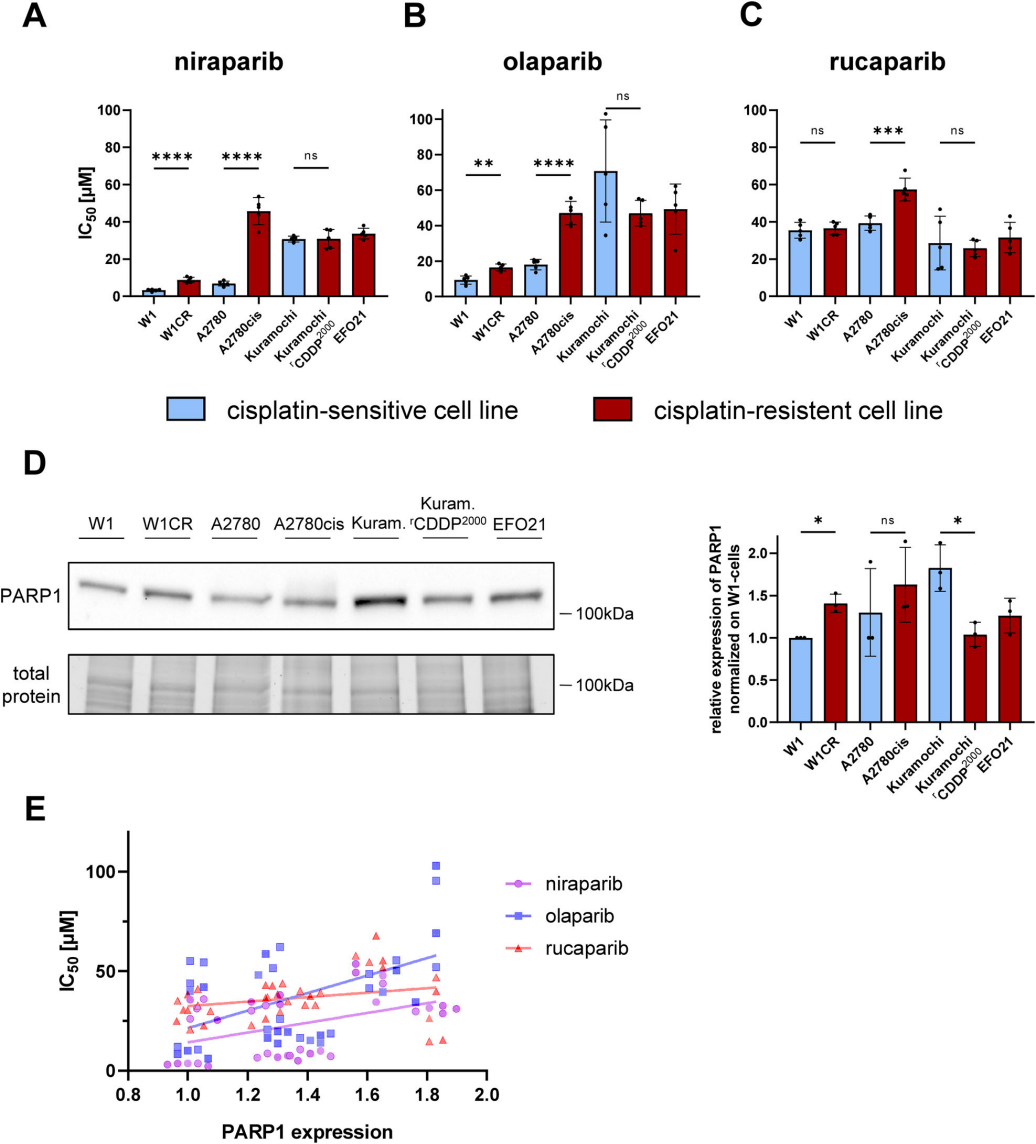
Cytotoxic activity of different approved PARPi in ovarian cancer cells and the impact of cisplatin resistance and PARP1 expresssion. **A–C** IC_50_-values of either niraparib, olaparib or rucaparib in the respective cell lines either cisplatin-sensitive (blue) or cisplatin-resistant (red). Mean ± SD (*n* = 5), for statistical analysis two-tailed unpaired *t*-test was performed, *P < 0.05, **P < 0.01; ***P < 0.001; ****P < 0.0001. **D** Representative Western Blot of PARP1 in the indicated cell lines. Histogram depicts relative protein expression of PARP1 normalized on W1 cells. Mean ± SD (*n* = 3), for statistical analysis two-tailed unpaired *t*-test was performed, *P < 0.05. **E** Correlation of the individual IC_50_-values and PARP1 expression in all investigated cell lines and the indicated PARPi.

First, we determined dose-response profiles to the approved PARPi niraparib, olaparib, and rucaparib in the ovarian cancer cell lines W1, A2780, and Kuramochi and their cisplatin-resistant sublines (W1CR, A2780cis, Kuramochi^r^CDDP^2000^) as well as EFO21, an intrinsically cisplatin-resistant cell line [21] used as an additional control (Fig. 1A-C). A2780 cells and EFO21 cells are known to display wt BRCA, while Kuramochi cells have been described to be BRCA2-mutant [22]. Since the BRCA genes had not been characterized in W1, we determined the BRCA1/2 status in W1 and W1CR cells. Both harbored a c.3858_3860delAAA (p.Lys1286del) mutation in Exon 11 of the BRCA2 gene, which is according to HSMD, OncoKB and ClinVar a variant of uncertain significance [23].

The parental cell lines differed in their relative resistance to the PARPi. W1 and A2780 cells were more sensitive to niraparib and olaparib compared to Kuramochi and EFO21 cells, but not to rucaparib (Fig. 1A–C). Moreover, the resistant cell lines displayed differing PARPi response profiles. A2780cis displayed cross-resistance to all three PARPi, W1CR cells to niraparib and olaparib but not to rucaparib, and Kuramochi^r^CDDP^2000^ was similarly sensitive to all three PARPi as Kuramochi (Fig. 1A–C).

Niraparib displayed the highest efficacy in most cell lines with IC_50_ values in the low micromolar range, but was also subject to cross-resistance in the W1 and A2780 cell pairs (Fig. 1A). The latter effect could also be seen in cells treated with olaparib. Its spectrum of activity is comparable to niraparib, albeit with lower potency (Fig. 1B). However, the cellular response to rucaparib was comparatively homogenous in the investigated cell lines giving hardly any signs of cross-resistance with cisplatin, except the A2780 cell pair. Rucaparib, on the other hand, showed the greatest potency in the Kuramochi cell pair and the intrinsic cisplatin-resistant EFO21 cells (Fig. 1C). Consequently, any deviations in the cell sensitivity to the various PARPi cannot be related to BRCA2 mutations in our cell lines. In particular, BRCA2-mutated W1 cell pair responds to a greater extent to all PARPi compared to the other cell lines, while Kuramochi cell pair, which is also BRCA2-mutated displays roughly 3-fold higher IC_50_-values of niraparib and olaparib.

Regarding the cellular levels of PARP1, which is one of the main targets of all PARPi [24, 25], only slight differences become evident (Fig. 1D). Plotting the expression of PARP1 and IC_50_ values of all investigated cell lines reveals the highest correlation for olaparib, followed by niraparib. In contrast, rucaparib activity seems to be independent from PARP1 levels (Fig. 1E). Together, the data indicate complex PARPi response patterns in cisplatin-resistant ovarian cancer sublines.

### No increased activity of PARPi and cisplatin in combination

**Fig. 2:**
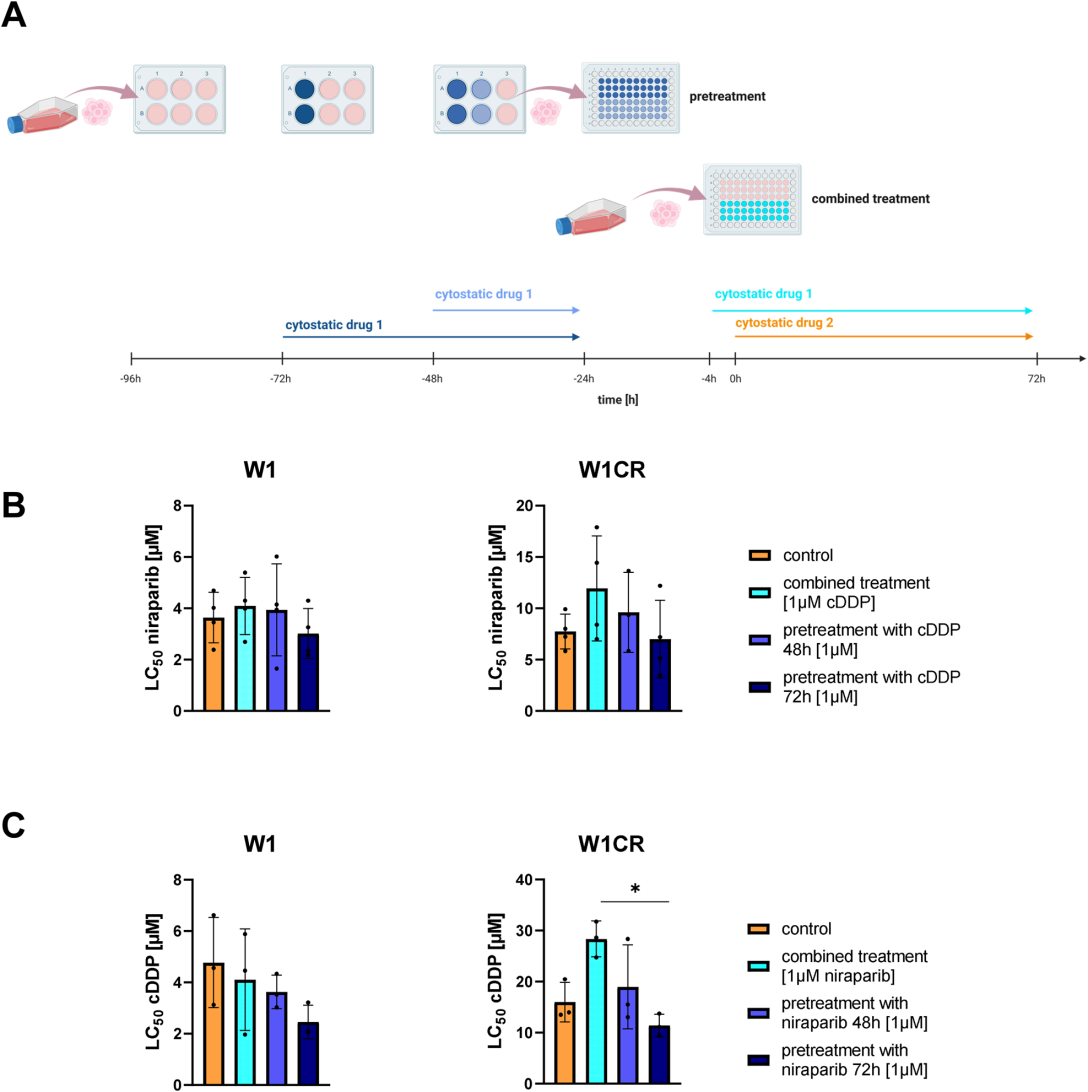
Combinational treatment of ovarian cancer cells with cisplatin and niraparib. **A** Graphical representation of experimental setup for **B** and **C**. **B,** **C** LC_50_ values of niraparib (**B**) or cDDP (**C**) in W1 and W1CR cells, pre- or combined treated with cDDP (**B**) or niraparib (**C**), both at 1 µM. Data represent means ± SD (*n* = 3). Statistical analysis for **B** and **C** was performed using paired *t*-tests. *P < 0.05.

Next, we investigated whether combined cisplatin and PARPi treatment results in an increased activity, using niraparib in W1 and W1CR cells as an example. Aiming to elucidate shared target activities of both drugs with impact on resistance formation, we tested cisplatin pretreatment, niraparib pretreatment in two different time ranges, and the addition of both drugs at the same time (Fig. 2A). However, the combinations did not result in a significantly increased activity (Fig. 2B, C).

### PARPi induce heterogeneous cell cycle dysregulations that serve as targets for sensitization

**Fig. 3:**
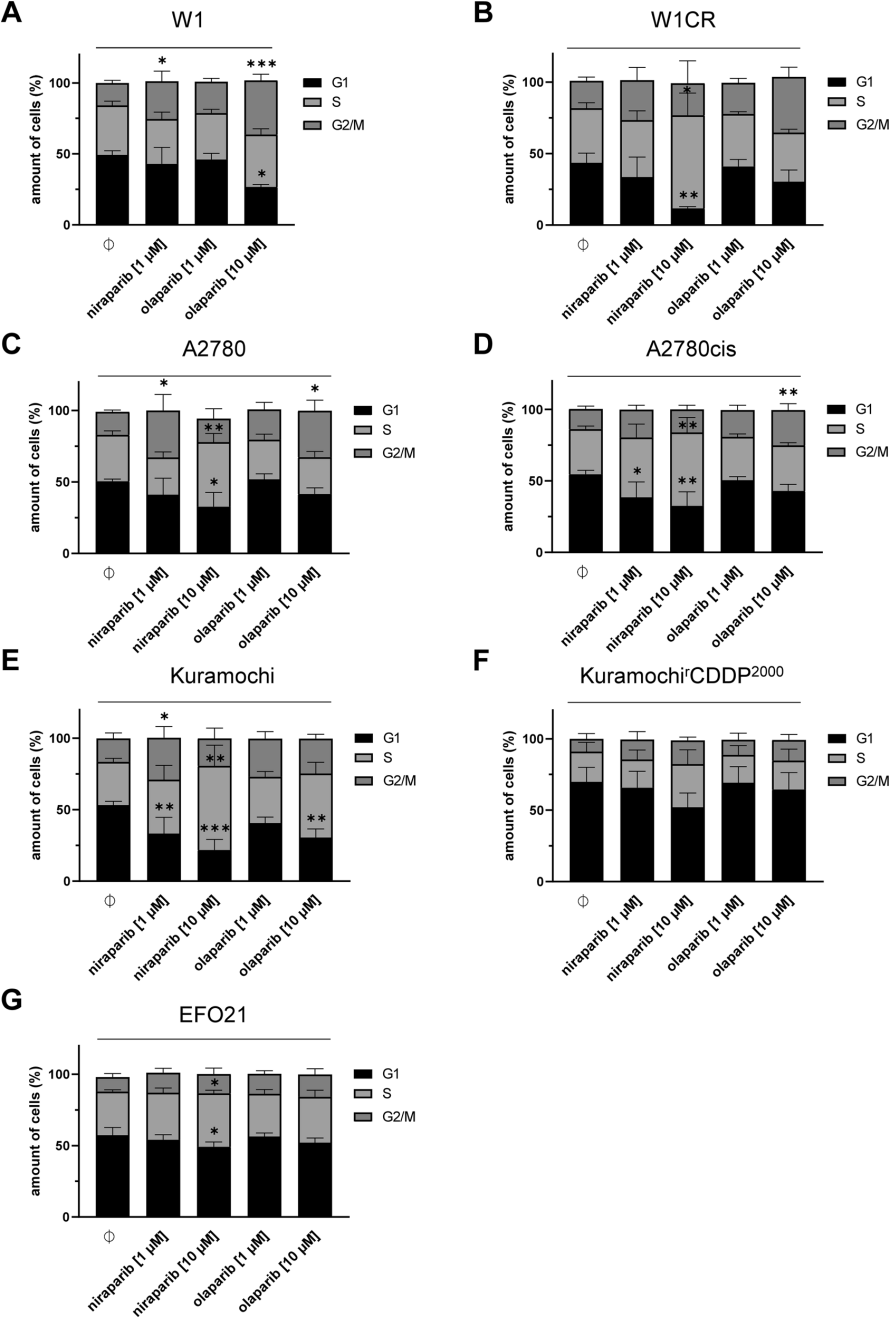
Impact of PARPi treatment on cell cycle regulation of ovarian cancer cells. **A–G** Cell cycle analysis of the indicated cell lines with either niraparib at 1 µM or 10 µM, or olaparib at 1 µM or 10 µM. Mean ± SD (*n* = 3) for the respective phase of the cell cycle. For statistical analysis, One-way ANOVA following Dunnett’s test was performed for each phase and cell line, *P < 0.05, **P < 0.01; ***P < 0.001; ****P < 0.0001.

To further investigate the different responses of the investigated cell lines to the PARPi, cell cycle analyses were performed. In light of the obvious independence of rucaparib from PARP1 expression, indicated above, only niraparib and olaparib were applied here at a low (1 µM) and a high (10 µM) concentration (Fig. 3). Due to the high niraparib sensitivity of W1, only niraparib at 1 µM could be used for this cell line.

Both PARPi affected the cell cycle differently. Niraparib generally induced more pronounced effects (Fig. 3). In the parental cell lines, niraparib caused an increase of G2/M-phase cells at the lower 1 µM dose and an arrest in the S-phase at the higher 10 µM dose (Fig. 3A, C, E). In the cisplatin-resistant sublines W1CR and A2780cis, and in EFO21 cells, niraparib 10 µM also induced an S-phase arrest, but no consistent changes at 1 µM (Fig. 3B, D, G). Kuramochi^r^CDDP^2000^ did not display statistically significant cell cycle changes in response to niraparib, despite a trend towards an accumulation of cells in S-phase (Fig. 3F). Compared to niraparib, olaparib caused less pronounced cell cycle changes.

**Fig. 4:**
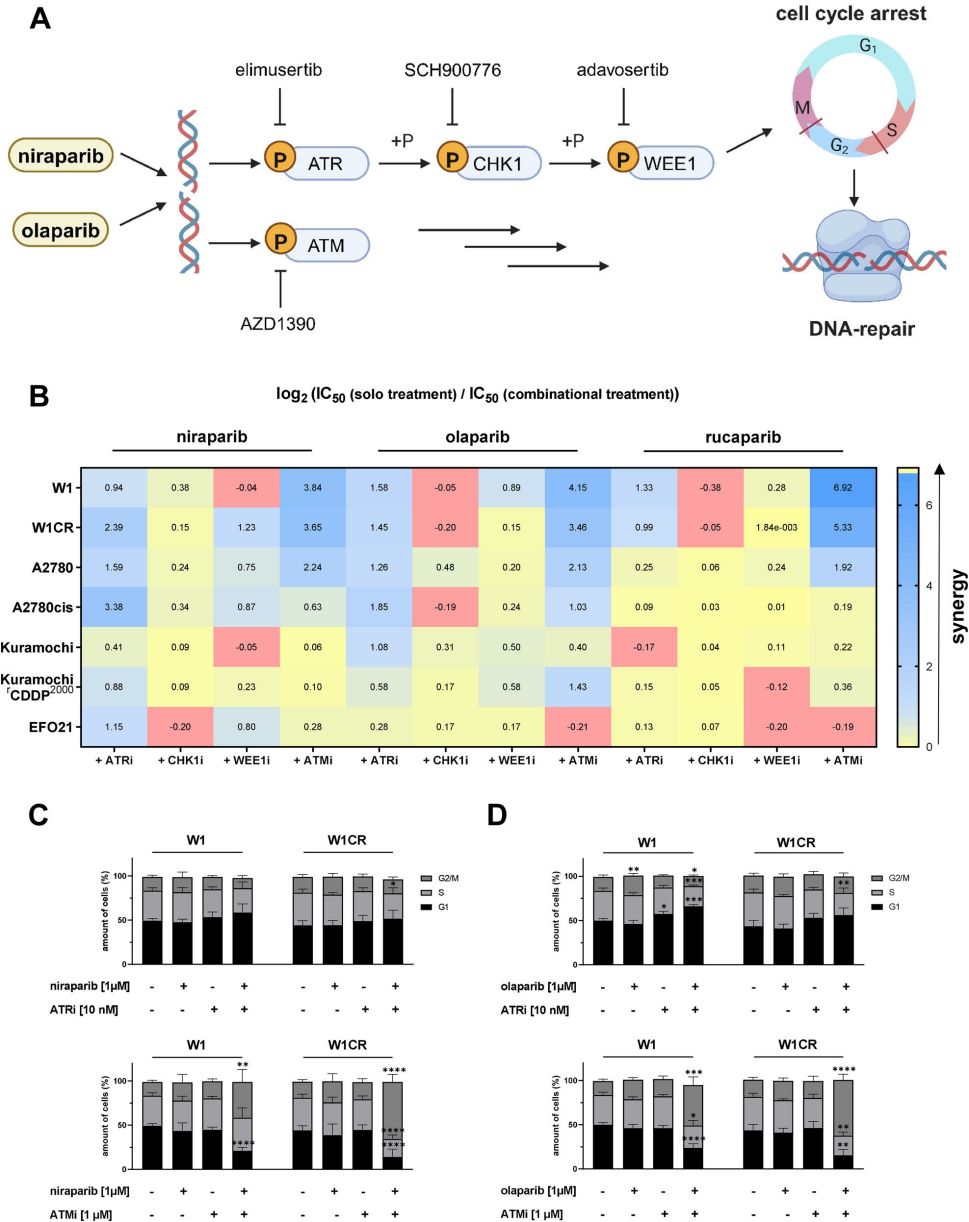
Insight into the role of DNA damage response pathway on PARPi activity. **A** Scheme for the investigated protein kinases that mediate cell cycle arrests and selective inhibitors of the indicated kinases used in this study. **B** Heatmap of combinational treatments using selective kinase inhibitors against either ATR, CHK1, WEE1 or ATM and niraparib, olaparib or rucaparib in the respective cell lines. The indicated data represent means of the log_2_ of the ratio of the IC_50_-values resulting from solo treatment and the IC_50_-values resulting from the respective combinational treatment (*n* = 3). **C,D** Cell cycle analysis upon treatment with the indicated PARPi alone, elimusertib (ATRi) or AZD1390 (ATMi) alone or the combination of the respective PARPi and ATRi or ATMi. Mean ± SD (*n* = 3) for the respective phase of the cell cycle. For statistical analysis One-way ANOVA following Dunnett’s test was performed for each phase and cell line, *P < 0.05, **P < 0.01; ***P < 0.001; ****P < 0.0001.

Next, we tested the impact of inhibitors of kinases involved in DNA-damage response signaling pathways (ATR, elimusertib; CHK1, SCH90076; WEE1, adavosertib; ATM, AZD1390), whose activation results in cell cycle arrest or mediates DNA repair [26], on PARPi activity (Fig. 4A). Sub-toxic doses of these kinase inhibitors were selected that had been determined in a previous study [21]. Overall, the ATR inhibitor elimusertib and the ATM inhibitor AZD1390 elicited the strongest increase in PARPi activity, including synergistic effects in a majority of cell lines (Fig. 4B, Supplement Fig. 1). Since rucaparib, again, shows different patterns of synergy which goes in line with its deviating dependency from PARP1 expression, we subsequently focused on niraparib and olaparib for a detailed analysis.

In a subsequent cell cycle analysis, a non-toxic concentration of the ATM inhibitor AZD1390 caused a profound G2/M cell cycle block in combination with non-toxic concentrations of niraparib (Fig. 4C) and olaparib (Fig. 4D) in W1 and W1CR cells. The ATR inhibitor elimusertib induced less pronounced and consistent effects (Fig. 4C, D).

### Collagen I-mediated PARPi resistance

**Fig. 5:**
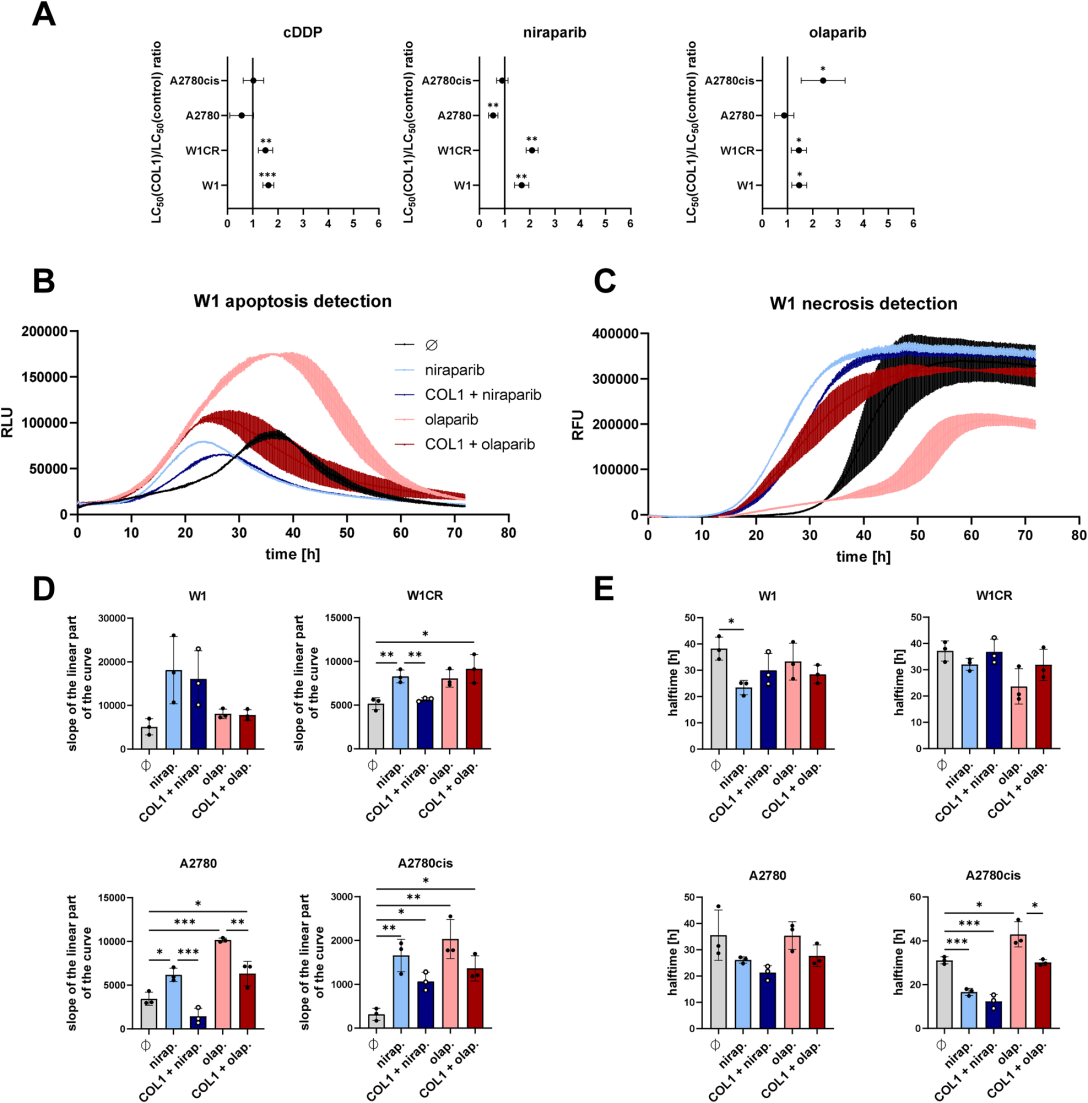
Impact of COL1 treatment of ovarian cancer cells on PARPi cytotoxicity. **A** Ratio of cDDP, niraparib and olaparib LC_50_ values between ovarian cells cultivated with and without COL1. This is shown for W1, W1CR, A2780 and A2780cis cells. Data represent means ± CI (*n* = 6). **B** Representative curve of live apoptosis detection in W1 cells upon the indicated treatments for 72 h. **C** Representative curve of live necrosis detection in W1 cells upon the indicated treatments for 72 h. **D** Histograms showing the slope of the initial linear part of the curves quantifying the kinetic of apoptosis induction. **E** Histograms showing the time required to reach half of the maximum fluorescence value. Both, apoptosis and necrosis were measured in parallel in the indicated cell lines treated with either 5 µM (W1), 15 µM (W1CR, A2780) or 40 µM (A2780cis) of the respective PARPi according to the cell viability data in Fig. 1. Mean ± SD (*n* = 3), for statistical analysis One-way ANOVA following Tukey’s test was performed, *P < 0.05, **P < 0.01; ***P < 0.001; ****P < 0.0001.

The ECM constituent collagen is known to mediate so-called CAM-DR against anti-cancer drugs, including cisplatin or doxorubicin [18, 27], but had not been investigated for its impact on cancer cell sensitivity to PARPi before. Hence, we next tested the effects of niraparib, olaparib, and cisplatin (serving as a control) on W1, W1CR, A2780, and A2780cis cells that were cultivated on collagen type I (COL1)-coated or uncoated wells (Fig. 5A, raw data shown in Supplement Fig. 2A).

COL1 protected W1 and W1CR cells from toxicity induced by all three drugs. However, cultivation of A2780 cells on COL1 did not reduce the efficacy of any of the compounds, and COL1 mediated resistance only to olaparib, but not to niraparib and cisplatin in A2780cis cells (Fig. 5A). This further confirms that the indicated cell lines display complex, varying phenotypes.

To get a further insight into the detailed effects of COL1 on attenuated cell toxicity, a live cell apoptosis and necrosis assay was applied, as exemplarily illustrated for W1 cells (Fig. 5B, C). The determination of apoptosis in niraparib- and olaparib-treated W1, W1CR, A2780, and A2780cis cells (Fig. 5D) and necrosis (Fig. 5E) also resulted in complex cell specific patterns. COL1 significantly reduced the induction of apoptosis of both PARPi in A2780 and A2780cis cells (Fig. 5D). Furthermore, there is a trend to increase the halftime to induce cell necrosis upon COL1 cultivation (Fig. 5E). Apart from this, there were no further consistent outcomes to interpret the activity of COL1 in this context.

### Targeting DNA-repair enzymes overcomes CAM-DR

**Fig. 6:**
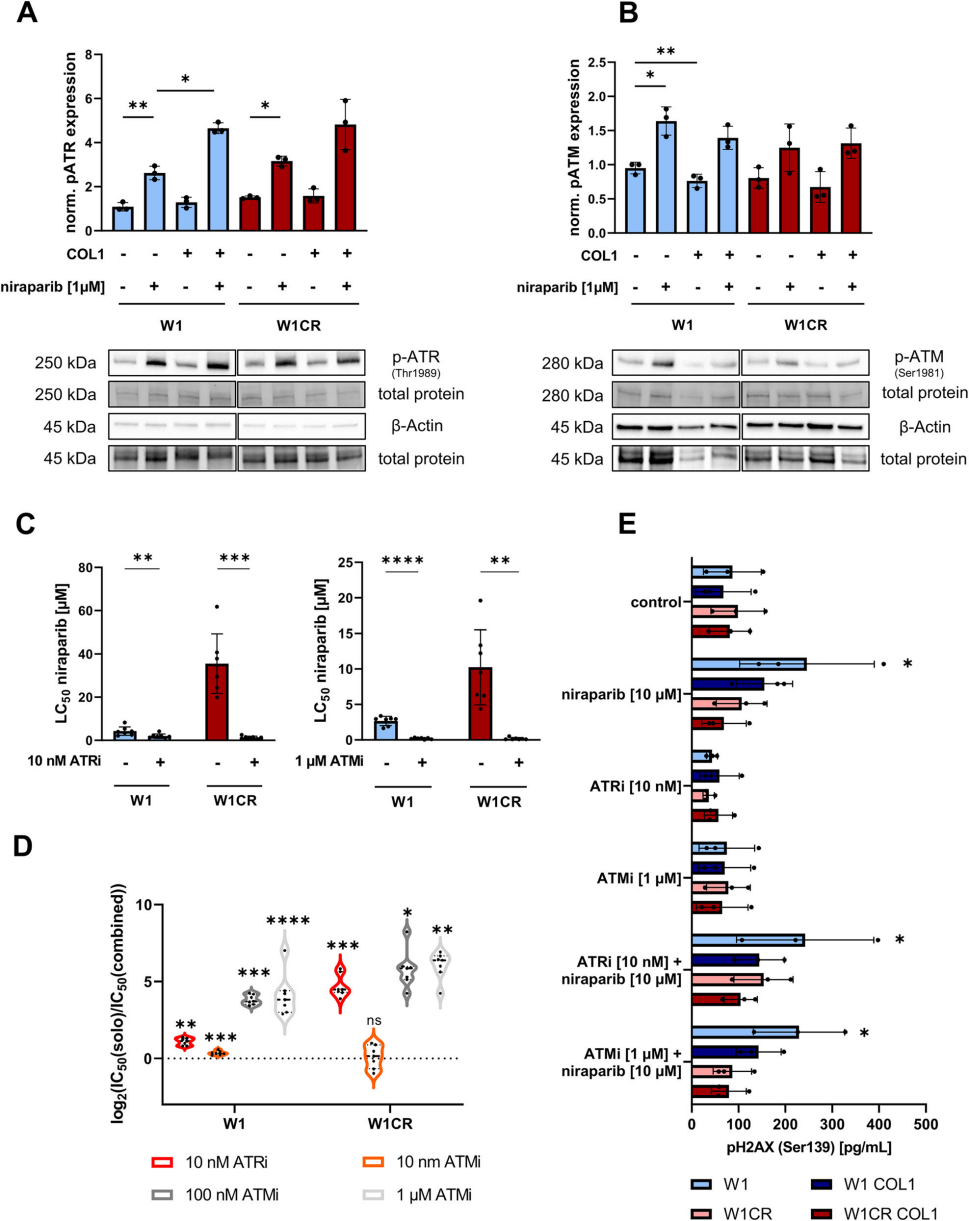
Insight into the COL1-mediated DNA damage repair signaling as target for sensitization strategies. **A**,**B** Impact of COL1 binding and niraparib at 1 µM on activation of ATR and ATM in W1 and W1CR cells after 24 h, analyzed by Western Blotting. **A** and **B** display the relative expressions of p-ATR (Thr1989) and p-ATM (Ser1981) normalized on untreated W1 cells. Protein bands of a representative Western Blot and the respective total protein bands of the stainfree visualization or the housekeeping protein band of *β*-actin are presented below the graphs. Data represent means ± SD (*n* = 3). To test for significance paired *t*-tests were applied for cultivation and unpaired *t*-tests for cell line comparison. *P < 0.05; **P < 0.01. **C** LC_50_ values of niraparib cytotoxicity in W1 and W1CR cells, treated with ATR or ATM inhibitors at the indicated concentrations. All cells were cultivated on COL1. Data represent means ± SD (*n* = 7). Statistical analysis was performed by paired *t*-tests. *P < 0.05; ** *P* < 0.01; *** *P* < 0.001; ****P < 0.0001. **D** Violin plot of log_2_ fold changes between cytotoxicity of solo (niraparib) and combined (niraparib + ATRi/ATMi) treated W1 and W1CR cells cultivated on COL1. Lines in violins represent median and quartiles (*n* = 7). Asterisks display the significance levels calculated in **C** and in Supplement Fig. 3. **E** Quantification of the protein amount of phosphorylated H2AX (Ser139) as a marker for damaged DNA under the indicated treatments in W1 and W1CR cells either cultivated on COL1 or not. Data represent means ± SD (*n* = 3). Statistical analysis was performed by One-Way ANOVA following Dunnett’s test. *P < 0.05.

To exploit the mechanisms of this COL1-mediated resistance, we investigated whether DNA repair enzymes were involved in this phenomenon. Therefore, we selected the W1 cell line and its sublines W1CR as they exhibited the most pronounced CAM-DR among all observed cell lines (Fig. 5A). Since the interaction with COL1 has no significant effect on PARP1 expression levels in W1 and W1CR cells (Supplement Fig. 2B), we focused on its impact on ATR and ATM expression which are the highlights from the sensitization approaches. For this purpose, the expression levels of phosphorylated ATR and ATM were characterized in both cell lines after an incubation of 24 h in absence or presence of COL1 and 1 µM niraparib (Fig. 6A, B). The Western blot data reveal that treatment with niraparib significantly increased ATR activity in both W1 and W1CR cells. In comparison, expression of phosphorylated ATM was elevated to a smaller extent. Interestingly, the cultivation on COL1 strongly enhanced this effect of niraparib treatment on ATR activation, whereas ATM activation was not further increased by contact with COL1 under niraparib treatment.

In the next step, we investigated whether the concomitant use of PARPi, with ATRi, or ATMi also results in a synergistic effect in cells cultivated on COL1. To clarify this issue, we focused on W1 and W1CR cells, as they form the most distinctive CAM-DR. Both cell lines were treated with 10 nM of ATRi and three different concentrations of ATMi (Fig. 6C and Supplement Fig. 3). The results display differences in the effectiveness of targeting the DDR pathway between inhibition of either ATR or ATM. Furthermore, differences in the responses of wildtype and platinum-resistant subtype became evident. While the treatment with 10 nM ATRi led to impressive sensitization to niraparib in both cell lines, the same concentration of ATMi did not affect PARPi effectiveness. On the other hand, increasing the concentration of ATMi to a still non-toxic concentration of 1 µM induced a sensitizing effect even surpassing the sensitization through 10 nM ATRi. Same doses of ATRi could not be applied due to cytotoxicity. To further summarize these data, the fold changes of IC_50_-values in combinational vs. solo treatment are shown as violin plot in Fig. 6D where the higher effectiveness of ATMi compared to ATRi becomes more clearly. In conclusion, the combination of targeting key DDR regulators and PARPi is more effective in W1CR cells, thereby encouraging its potential for overcoming CAM-DR. Especially the inhibition of ATR has a greater impact in this cisplatin-resistant subtype compared to wildtype W1 cells. To further elucidate the impact of COL1 on DNA damage, we performed an ELISA to quantify the amount of phosphorylated H2AX, which serves as a marker of DNA damage. The data show a significant increase of pH2AX upon treatment with niraparib in W1 cells, whereas W1CR cells seem unaffected reflecting the cross-resistance between cisplatin and niraparib (Fig. 6E). In addition, cultivation on COL1 slightly reduces DNA damages in both cell lines confirming the role of the ECM protein in mediating DNA repair. Neither the inhibition of ATR nor ATM in the solo-treated samples at sub-toxic concentrations had an impact on pH2AX. A slight increase in DNA damage was observed in both W1CR cell samples when ATRi and niraparib were combined. The inhibition of ATM showed the opposite effect in these cells. In contrast, W1 cells did not show any change in their amount of pH2AX through combinational treatments compared to solo niraparib treatment (Fig. 6E).

## Discussion

The introduction of PARPi into the guideline-based therapy of ovarian cancer has expanded the clinically available treatment opportunities. However, resistance development remains the major therapy-limiting factor [28], in particular since PARPi are currently only used for platinum-sensitive ovarian cancer [3]. Since there is limited information available on the efficacy of PARPi against cisplatin-resistant ovarian cancer cells, we here tested the PARPi niraparib, olaparib, and rucaparib in a panel of cisplatin-adapted ovarian cancer cell lines.

Cisplatin resistance was in some, but not all cisplatin-resistant sublines associated with cross-resistance to PARPi. While W1CR cells displayed cross-resistance to niraparib and olaparib, W1 and W1CR cells were similarly sensitive to rucaparib. Cell cycle investigations further demonstrated that the investigated PARPi induced complex cell line-specific responses. Notably, niraparib did not increase cisplatin activity in W1 and W1CR cells and vice versa. Additionally, the expression of PARP1 is not suitable as an indicator for responsiveness to PARPi, since the deviations of PARP1 levels among the cell lines are rather low. These data also demonstrate that resistance formation against cisplatin has no uniform consequence for PARP1 expression. On the other hand, PARP1 expression correlates with resistance to olaparib and to niraparib to a lower amount. Rucaparib acts independently from PARP1 levels indicating that its cytotoxic activity is driven by off-target effects [29, 30].

The investigation of the effects of a panel of DDR inhibitors (including ATR, ATM, CHK1, and WEE1 inhibitors) for their potential to sensitize the indicated cell lines of this project to PARPi, resulted again in very complex activity profiles. Nevertheless, the ATR inhibitor elimusertib and the ATM inhibitor AZD1390 demonstrated the biggest potential to increase the PARPi-mediated anti-cancer effects and to partly overcome cross-resistance phenomena.

Although environmental-mediated resistance phenomena, such as CAM-DR are known to reduce cancer cell sensitivity to DNA damaging drugs, including cisplatin [18, 27], PARPi activities had not yet been considered in this context. Cultivation of W1 and W1CR on COL1 reduced the sensitivity of these cell lines to the PARPi niraparib and olaparib. In contrast, COL1 did not decrease the PARPi sensitivity of A2780 cells and mediated resistance only to olaparib, but not to niraparib in A2780cis cells. However, differences between an endpoint cell viability detection and data of the kinetic detection of apoptosis and necrosis formation became evident. These findings further underline the complexity of the phenotypes of the investigated cell lines but also show that cell adhesion-mediated effects can contribute to PARPi resistance. In this context, Western Blot data indicate a potential mechanistic link between the activity of DDR signaling and the observed CAM-DR as we detected a niraparib-mediated upregulation of ATR and ATM activity, which was further enhanced by COL1 in case of ATR. Consequently, the ATR inhibitor elimusertib, but also the ATM inhibitor AZD1390 interfered with cell adhesion-mediated PARPi resistance. To further elucidate the role of both kinases and COL1 on regulating DNA damage, we found that the ECM-protein is able to reduce the amount pH2AX under PARPi treatment. Nevertheless, additional inhibition of ATR or ATM and PARPi treatment did not significantly increase this DNA damage marker showing that multiple kinases in parallel are involved in the phosphorylation of H2AX. Finally, these data show that not only accumulated DNA damage comes into play when ATRi or ATMi are combined with PARPi, but also other cellular processes are affected under these conditions that contribute to resistance against PARPi, which requires further investigations.

Taken together, our findings revealed complex, cell line-specific response profiles to the investigated drugs. These findings are in line with other studies investigating drug-resistant cancer cell lines, including those, in which the same cell line was repeatedly adapted to the same drug in multiple experiments [31–34], and also with the complex evolutionary processes in cancer cells from lung cancer patients [35–37]. Despite this complexity, we identified ATM and ATR inhibitors as promising agents that can increase the efficacy of PARPi against ovarian cancer cells, including those with acquired cisplatin resistance. Moreover, we demonstrate for the first time that cell adhesion-mediated resistance can affect the efficacy of PARPi and that ATR and ATM inhibitors also have the potential to alleviate this cancer microenvironment-associated form of resistance.

## Materials and Methods

### Cell culture

All examined human ovarian cancer cell lines were cultivated in RPMI 1640 medium (PAN Biotech GmbH, Aidenbach, Germany) at 37 °C and 5% CO_2_. The medium was supplemented with 10% fetal calf serum (FCS) (PAN Biotech GmbH), 100 IU/ml penicillin and 100 µg/ml streptomycin. The absence of a mycoplasma contamination was confirmed every month. W1 cells and its cisplatin-resistant variant W1CR were generously provided to us by Dr. R. Januchowski (Zielona Gora, Poland) and first described in [38]. A2780 cells and their cisplatin-resistant subtype A2780cis were obtained from ECACC, UK (No. 93112519; No.93112517-A2780cis). The Kuramochi cell line was obtained from JRCB (Osaka, Japan), while its cisplatin-resistant subtype Kuramochi’CDDP^2000^ was derived from The Resistant Cancer Cell Line (RCCL) collection [39]. The cell line EFO21 was received from DSMZ (Braunschweig, Germany). To maintain the cisplatin-resistance in the sublines W1CR, A2780cis and Kuramochi^r^CDDP^2000^, the cells were regularly treated with 3000 ng/ml cisplatin (Sigma-Aldrich GmbH, Steinheim, Germany). To avoid adulteration by cisplatin, the following passage of the cells was not used for experiments.

### BRCA1/2 gene mutation analysis in W1 and W1CR cells

DNA from W1 and W1CR cells for the subsequent genetic testing was extracted and purified using the Monarch^®^ Genomic DNA Purification Kit from New England Biolabs Inc. (Ipswich, MA, USA). The mutation analysis was performed by next-generation sequencing (NGS) on a Miniseq sequencing instrument (Illumina, San Diego, CA, USA) using a custom QIAseq-BRCA1/2plus-panel (QIAGEN, Hilden, Germany). The generated data was analyzed via the Biomedical Genomics Workbench (QIAGEN, Hilden, Germany). To interpret the relevance of the detected mutation three databases (Human Somatic Mutation Database, OncoKB™, ClinVar) were applied. The analysis was conducted by Genopath GbR (Bonn, Germany).

### Cell viability assay

To determine the cytotoxicity of the PARPi niraparib, olaparib, and rucaparib (purchased from Hölzel Diagnostika Handels GmbH, Cologne, Germany) on ovarian cancer cells, a colorimetric cell viability assay using MTT (3-(4,5-dimethylthiazol-2-yl)-2,5-diphenyltetrazolium bromide), (BioChemica, Applichem GmbH, Darmstadt, Germany) was applied. Cisplatin was used as a cytotoxic control. To analyze the involvement of the ECM, collagen-coated plates were used. For combined treatments, the cells were pretreated with inhibitors against ATR (elimusertib), ATM (AZD1390), CHK1 (SCH900776), and WEE1 (adavosertib), (all purchased from Hölzel Diagnostika Handels GmbH, Cologne, Germany), cisplatin or niraparib for 4, 48 or 72 h at non-toxic concentrations.

All cell lines were transferred in triplicates at a total volume of 100 µL in 96-well plates (Sarstedt AG & Co, Nümbrecht, Germany). W1, W1CR, A2780 and A2780cis cells were seeded at a density of 10,000 cells/well, while EFO21, Kuramochi and Kuramochi^r^CDDP^2000^ cells were at a density of 5000 cells/well. After 24 h, cells were treated with a half logarithmic dilution series of either cisplatin, niraparib, olaparib or rucaparib (10^-4^ to 10^-8 ^M) and incubated for 72 h. At the end of the incubation period, 20 μL of an MTT solution (5 mg/mL) was added in each well and incubated for 1 h at 37 °C and 5% CO_2_. After removing the supernatant, formed formazan crystals were solubilized in 200 μL DMSO per well. Finally, absorption was analyzed at 570 nm using a plate reader (Thermomultiscan EX, Thermo, Schwerte, Germany). Background absorption at 690 nm was subtracted. Normalized sigmoidal dose response curves with variable hill slopes were generated and IC_50_ values were calculated by non-linear regression using a four-parameter logistic equation using GraphPad Prism 8 (GraphPad Software, San Diego, CA, USA). For statistical analysis, IC_50_-values were converted in pIC_50_-values.

### Western blot

Cells were lysed using cell extraction buffer (Bender MedSystems GmbH, Vienna, Austria) and total protein quantification was performed by Pierce™ BCA Protein Assay Kit (Thermo Fisher Scientific Inc., Waltham, MA, USA). After SDS-PAGE with 20 µg protein per lane using stain-free gels (Bio-Rad Laboratories GmbH, Munich, Germany), proteins were transferred to PVDF-membranes via Trans-Blot^®^ Turbo™ system (Bio-Rad Laboratories). After blocking unoccupied binding sites with a 5% solution of non-fat dry milk powder in TBS-T for 1 h, membranes were incubated with various primary-antibody solutions overnight. Therefore, we used rabbit anti-pATR (Thr1989) (#30632S), rabbit anti-pATM (Ser1981) (#13050 T) (purchased from Cell Signaling Technology, Frankfurt am Main, Germany), mouse anti-PARP1 (#sc-56197), mouse anti-α-tubulin (#sc-8035), (purchased from Santa Cruz Biotechnology, Heidelberg, Germany) and mouse anti-GAPDH (#T0004; GeneTex, Irvine, CA, USA) diluted in TBS-T containing 1% BSA and 0.05% sodium azide. The next morning, the membranes were incubated with a 5% non-fat dry milk solution containing goat anti-rabbit or anti-mouse IgG kappa binding IgG HRP-conjugated (Santa Cruz Biotechnology) diluted in TBS-T for 2 h. For visualization, we used the Clarity Western ECL substrate chemiluminescence kit (Bio-Rad Laboratories) and the ChemiDoc XRS+ imaging acquiring system (Bio-Rad Laboratories). For quantification and analysis, the band intensities were normalized to total protein expression using ImageLab software v 6.0 (Bio-Rad Laboratories). In addition, housekeeping proteins ran on the same membranes as further control.

### Cell cycle analysis

Cells were transferred in culture flasks at a density of 2 × 10^6^ cells and cultivated at 37 °C and 5% CO_2_ until the next day. Afterwards, cells were treated with either 1 or 10 µM of niraparib or olaparib or with PBS as a control. For combined treatments the ATRi elimusertib at 10 nM or the ATMi AZD1390 at 1 µM were used together with the lower dose of 1 µM of the respective PARPi. After an incubation period of 24 h, the assay was performed as described in [40]. To analyze the cell cycle we used Guava® easyCyte HT 11 Flow Cytometer (Luminex Corporation, Austin, TX, USA) and FlowJo™ v10.5.3 Software (BD Life Sciences, Franklin Lakes, NJ, USA).

### Collagen coating

For cell viability assays microplates were coated with 10 µg/cm^2^ COL1 from rat tail (Corning^®^, Bredford, PA, USA). Collagen dissolved in DPBS was transferred into plates and incubated for 1 h at 37 °C. After that the supernatant was removed and the COL1 coat was washed with DPBS.

### Real time apoptosis and necrosis assay

Cells were seeded at a density of 10,000 cells/well in a partially COL1-coated, white 96-well plate and cultivated at 37 °C and 5% CO_2_ in RPMI 1640 medium without phenol red overnight. The next day, W1 cells were treated with either niraparib or olaparib at a final concentration of 5 µM. W1CR and A2780 cells were treated with 15 µM of niraparib or olaparib and A2780cis cells with 40 µM of the respective PARPi. 100 µM cisplatin was used as a positive control, DPBS as a non-compound control and RMPI 1640 medium as a no-cell control. Subsequently, mixed 2× detection reagent of the RealTime Glo™ Annexin V Apoptosis and Necrosis assay kit (Promega Corporation, Madison, WI, USA) were added resulting in a total volume of 200 µL/well. After shaking the microplate for 30 seconds, the cells were analyzed at 37 °C at 10 min intervals for 72 h. During this period luminescence and fluorescence (485 nm extinction range, 525 nm emission range) were measured alternately by Tecan Spark microplate reader (Tecan Trading AG, Männedorf, Switzerland). Background measurement was subtracted and kinetic curves were created with GraphPad Prism 8 (GraphPad Software). The respective slopes of the resulting luminescence curves were used to compare the kinetic of apoptosis-initiation.

### Intracellular ELISA

To determine the intracellular amount of phosphorylated H2AX as a marker for damaged DNA, an intracellular ELISA assay (#DYC2288-2; Bio-Techne GmbH, Wiesbaden, Germany) was performed according to manufactures instructions. Briefly, 5 × 10^5^ cells were seeded into 6-well plates (Sarstedt AG & Co) either coated with COL1 (Corning^®^) at 10 µg/cm^2^ or uncoated and incubated overnight at 37 °C and 5% CO_2_. The next day, cells were treated with either niraparib at 10 µM, elimusertib at 10 nM, AZD1390 at 1 µM, a combination of niraparib and elimusertib or niraparib and AZD1390 or PBS as negative control. After 24 h of incubation, cell pellets were collected, lysed and the protein amount was determined via BCA assay as previously described. Afterwards, a total protein amount of 40 µg of each sample was pipetted in technical duplicates into a 96-well plate and the assay was performed as described in the manual.

### Statistical analysis

The statistical analysis was performed using GraphPad Prism 8 (GraphPad Software). The resistance factors shown in Fig. 5. are displayed as mean with 95% CI, while the violin plots show medians with quartiles. All other data are represented as means ± SD. For the comparison of two groups, we used an unpaired t-test. To test for significant changes through COL1-binding, we use a paired t-test. For comparisons between more than two groups, one-way ANOVA following Tukey’s test was performed. To check for significances between several groups and control groups, one-way ANOVA following Dunnett’s test was applied (asterisks indicate *P < 0.05; **P < 0.01; ***P < 0.001; ****P < 0.0001).

## Supplementary information

Supplementary Figures 1-3

Related Manuscript File

## Data Availability

All relevant data curated for this study are presented in the manuscript or in the supplementary data files. Raw data used for this study will be provided by the corresponding author upon reasonable request. The full length uncropped original western blots are shown in the Supplementary Material.
