# Supplementary material for: Increased efficacy of PARP inhibitors against cisplatin-sensitive and -resistant ovarian cancer cells mediated via ATR and ATM inhibition
Source: Cell Death Discov. 2025 Oct 6;11:438. doi: 10.1038/s41420-025-02740-1 (PMC12501025; doi:10.1038/s41420-025-02740-1)
Supplement: Supplementary file 1 — Supplementary Figures 1-3 [file 41420_2025_2740_MOESM1_ESM.pdf]

# W1

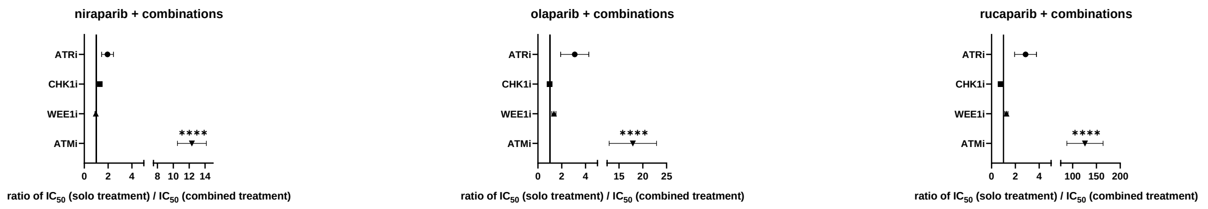


# W1CR

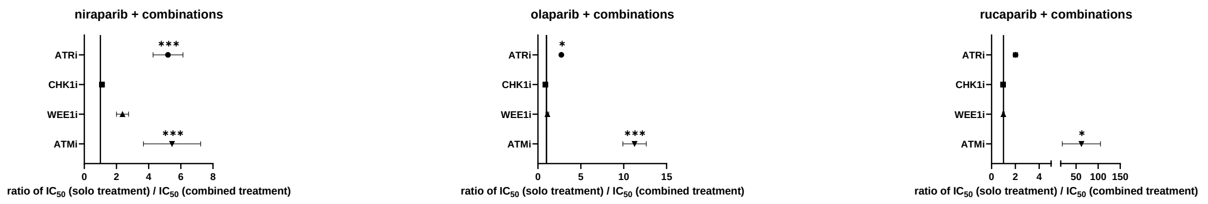


# A2780

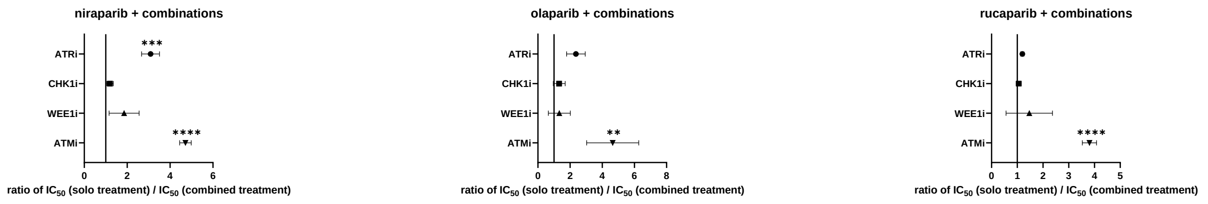


# A2780cis

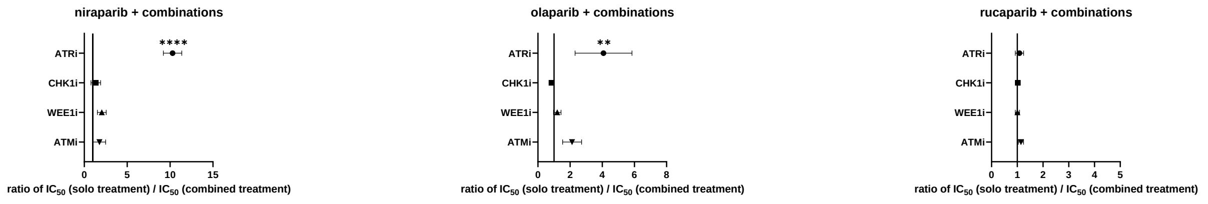


# Kuramochi

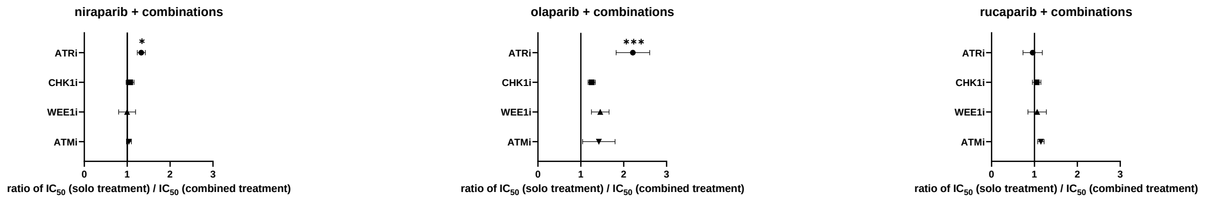


# Kuramochi$^{r}$CDDP$^{2000}$

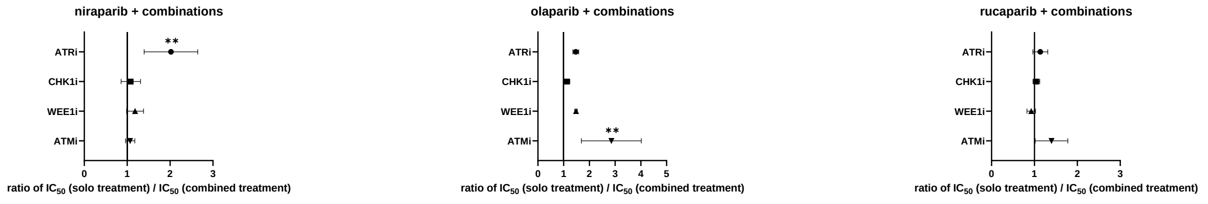


# EFO21

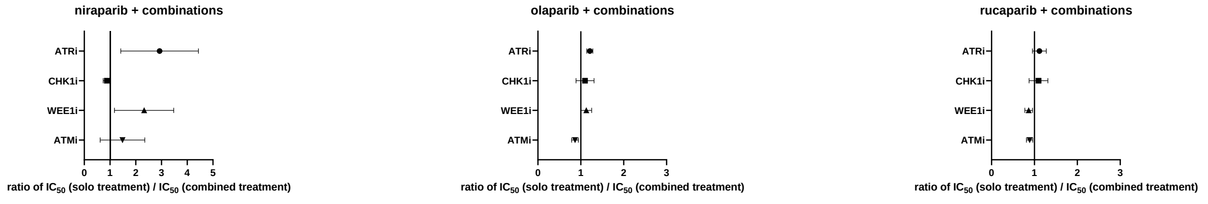

Supplement Figure 1: Ratio of $IC_{50}$ values of either niraparib, olaparib or rucaparib as solo treatment or in combination with either ATRi elimusertib [10 nM], CHK1i SCH900776 [1 µM], WEE1i adavosertib [10 nM] or ATMi AZD1390 [1 µM] in the indicated cell lines. Data are according to the heatmap shown in Fig. 4b and represent the means ± SD of at least three independent experiments (n = 3). Statistics were performed as One-way ANOVA following Dunnett's test using the solo treatment as control value. *$P < 0.05$, **$P < 0.01$, ***$P < 0{:}001$, ****$P < 0{:}0001$.

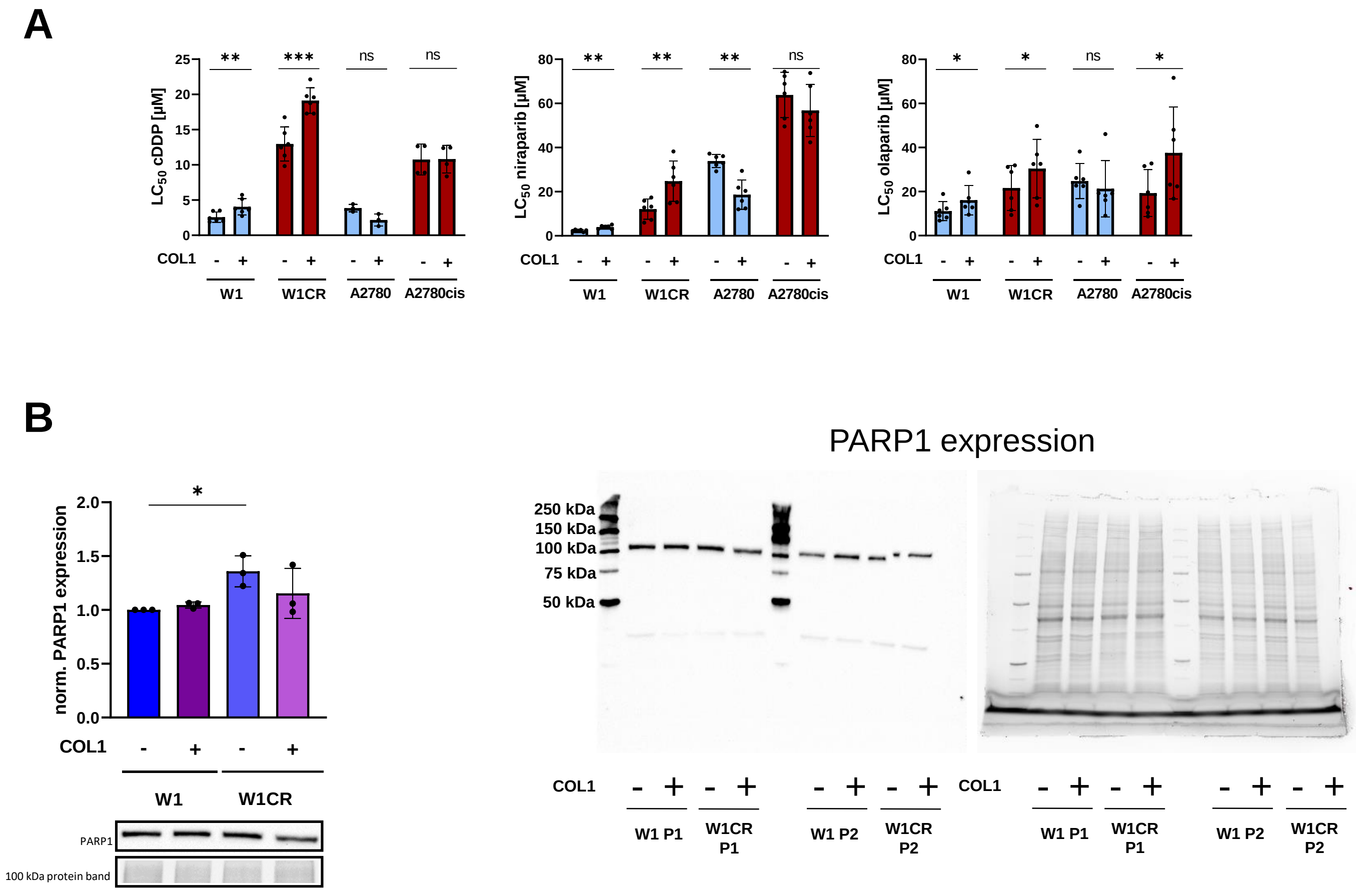


Supplement Figure 2: **A** $IC_{50}$-values of either cDDP, niraparib or olaparib in the W1 and A2780 cell pair in the absence or presence of COL1 (cisplatin-sensitive cells in blue and cisplatin-resistant in red). Mean ± SD (n = 6). Statistical analysis was performed using paired *t*-tests, *$P < 0.05$, **$P < 0.01$; ***$P < 0.001$. **B** Effect of COL1 binding on PARP1 expression in W1 and W1CR cells. Histogram displays the relative protein expression of PARP1 normalized on untreated W1 cells. Protein bands of representative Western Blots are shown below the histogram. Data represent means ± SD (n = 3). To test for significance paired *t*-tests were applied for cultivation and unpaired *t*-tests for cell line comparison. *$P < 0.05$

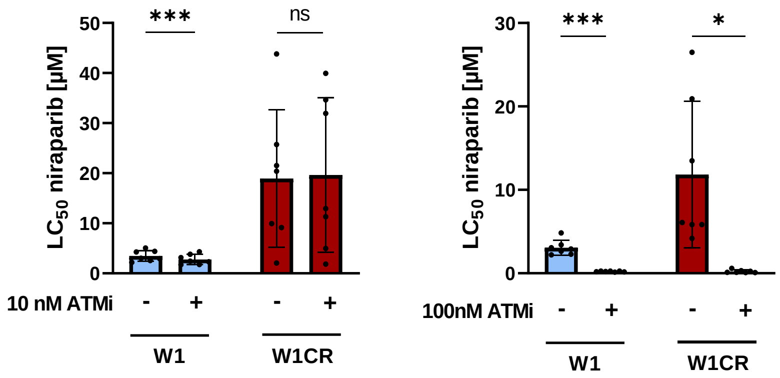


Supplement Figure 3: $LC_{50}$ values of niraparib cytotoxicity in W1 and W1CR cells, treated with ATR or ATM inhibitors at the indicated concentrations. All cells were cultivated on COL1. Data represent means ± SD (n = 7). Statistical analysis was performed by paired *t*-tests. * $P$ <0.05; ** $P$ <0.01; *** $P$ <0.001.
