# Supplementary material for: Increased efficacy of PARP inhibitors against cisplatin-sensitive and -resistant ovarian cancer cells mediated via ATR and ATM inhibition
Source: Cell Death Discov. 2025 Oct 6;11:438. doi: 10.1038/s41420-025-02740-1 (PMC12501025; doi:10.1038/s41420-025-02740-1)

Fig. 1D PARP1 expression after 24h

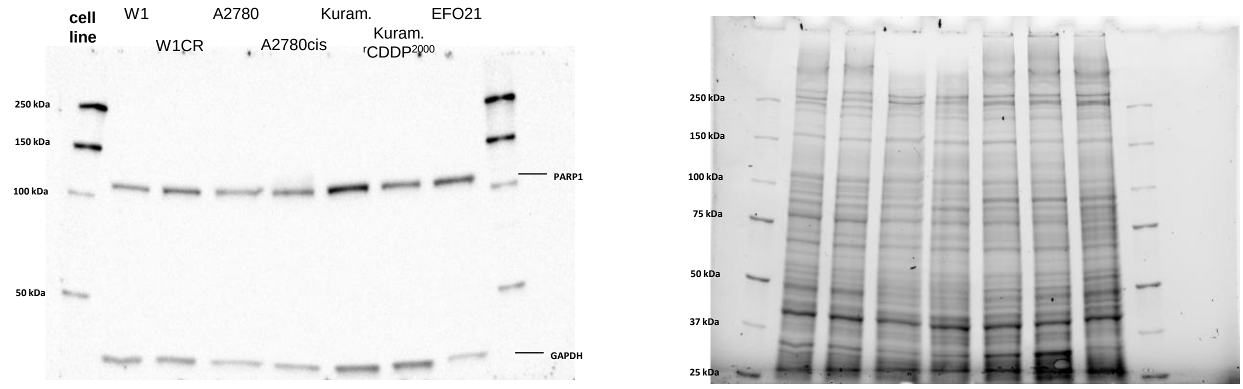

Fig. 6A Phospho-ATR expression after 24h

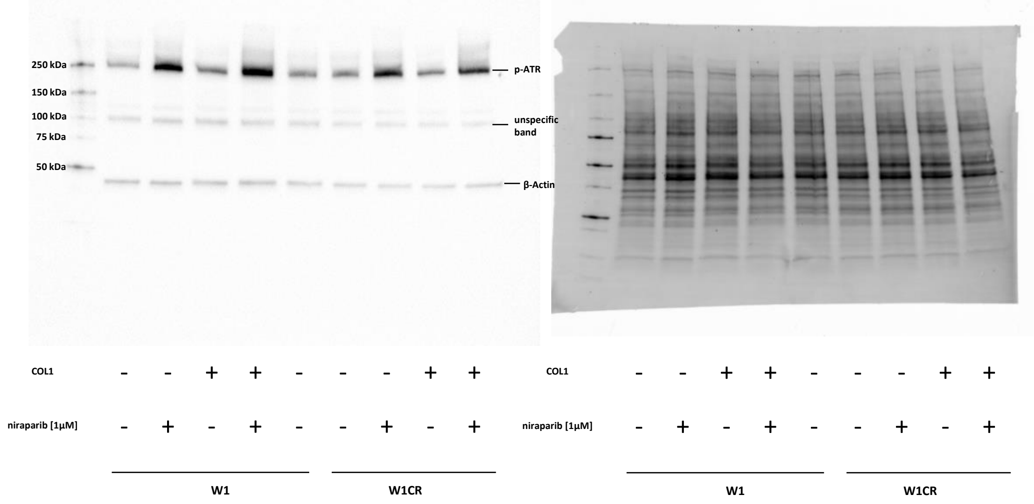

Fig. 6B Phospho-ATM expression after 24h

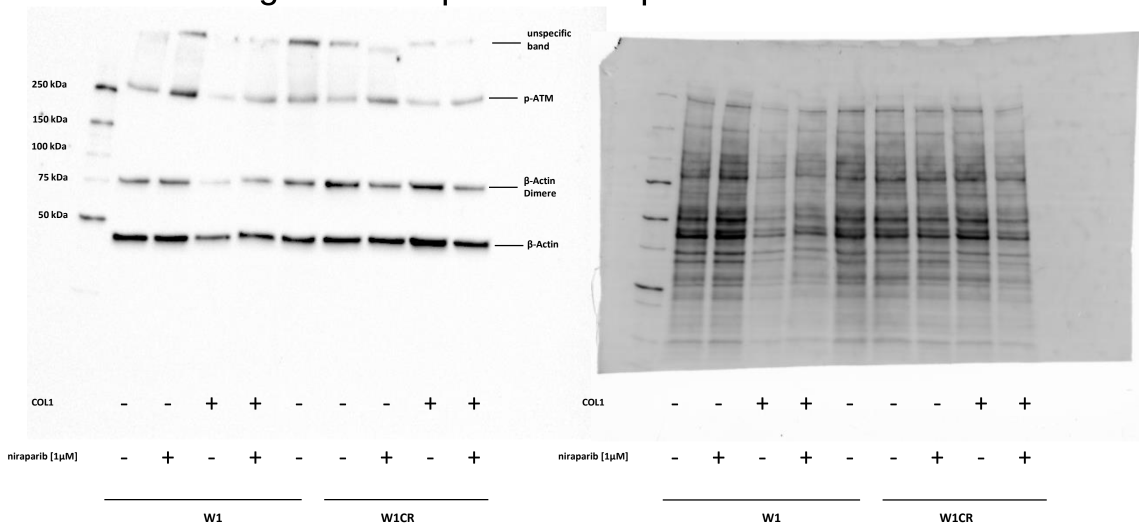

Supplement: Supplementary file 2 — Related Manuscript File [file 41420_2025_2740_MOESM2_ESM.pdf]
